# Silencing of hepcidin enforces the apoptosis in iron-induced human cardiomyocytes

**DOI:** 10.1186/1745-6673-9-11

**Published:** 2014-03-18

**Authors:** Yao-Peng Hsieh, Ching-Hui Huang, Chia-Ying Lee, Ching-Yuang Lin, Chia-Chu Chang

**Affiliations:** 1 Division of Nephrology, Department of Internal Medicine, Changhua Christian Hospital, 135 Nanhsiao St., Changhua 500, Taiwan; 2 Division of Cardiology, Department of Internal Medicine, Changhua Christian Hospital, Changhua, Taiwan; 3 Division of Pediatric Nephrology, China Medical University Hospital, Changhua, Taiwan; 4 Clinical Immunological Center, China Medical University Hospital, No. 2, Yuh-Der Road, Taichung, Taiwan; 5 Graduate Institute of Clinical Medical Science, College of Medicine, China Medical University, Taichung, Taiwan; 6 Program for Aging, China Medical University, Taichung, Taiwan; 7 School of Medicine, Chung Shan Medical University, Taichung, Taiwan

**Keywords:** Hepcidin siRNA, GATA-4, Apoptosis, Human cardiomyocytes

## Abstract

**Background:**

Iron is essential not only for erythropoisis but also for several bioenergetics’ processes in myocardium. Hepcidin is a well-known regulator of iron homeostasis. Recently, researchers identified low hepcidin was independently associated with increased 3-year mortality among systolic heart failure patients. In addition, our previous *in vivo* study revealed that the left ventricular mass index increased in chronic kidney disease patients with lower serum hepcidin. We hypothesize that hepcidin interacts with the apoptotic pathway of cardiomyocytes during oxidative stress conditions.

**Methods:**

To test this hypothesis, human cardiomyocytes were cultured and treated with ferrous iron. The possible underlying signaling pathways of cardiotoxicity were examined following knockdown studies using siRNAs of hepcidin (siRNA1 was used as a negative control and siRNA2 was used to silence hepcidin).

**Results:**

We found that ferrous iron induces apoptosis in human cardiomyocytes in a dose-dependent manner. This iron-induced apoptosis was linked to enhanced caspase 8, reduced Bcl-2, Bcl-xL, phosphorylated Akt and GATA-4. Hepcidin levels increased in human cardiomyocytes pretreated with ferrous iron and returned to non-iron treated levels following siRNA2 transfection. In iron pretreated cardiomyocytes, the siRNA2 transfection further increased caspase 8 expression and decreased the expression of GATA-4, Bcl-2, Bcl-xL and phosphorylated Akt than iron pretreatment alone, but caspase 9 levels remained unchanged.

**Conclusions:**

Our findings suggest that hepcidin can rescue human cardiomyocytes from iron-induced apoptosis through the regulation of GATA-4/Bcl-2 and the extrinsic apoptotic pathway.

## Introduction

Iron is essential not only for erythropoiesis but also for several bioenergetic processes in the myocardium [1]. Cardiac muscle iron overload leads to systolic and diastolic abnormalities, cardiac hypertrophy, and thus myocardial dysfunction in animal models [2]. At the molecular level, excess extracellular iron in heart muscle is thought to induce the formation of reactive oxygen species (ROS), such as superoxide and hydroxyl radicals via the Haber Weiss and Fenton reactions. The overproduction of these ROS, in turn, increases the susceptibility to cardiac injury and cell death [2]. In contrast, chronic iron deficiency may, by itself, reduce exercise capacity and cause ultrastructural alterations in cardiomyocytes [3]. Indeed, Dong et al. found that in iron-deficient rats, heart weight and left ventricle dimension were increased, as well as mitochondrial swelling and sarcomere disorganization, which may collectively lead to cardiac hypertrophy [4].

Hepcidin, a recently discovered 25-amino acid peptide that contains four disulfide bonds, was first identified in human urine and plasma [5]. The pivotal role of hepcidin in iron homeostasis was established in transgenic mouse models and in human diseases that result because of either its excess or deficiency [6,7]. Further studies found that hepcidin regulates iron homeostasis by binding to and inducing the internalization and degradation of ferroportin, the sole cellular iron efflux channel in iron-transporting cells [8]. Elevated hepcidin inhibits the release of stored iron from macrophages and hepatocytes into the circulation. The net effect of which is greater intracellular iron storage, along with less iron dietary absorption and circulating availability [9].

However, an increasing number of studies show that iron deficiency itself inhibits the expression of hepcidin and its release into the circulation [10-12]. Weber and colleagues reported that hepcidin levels in the lower tertile were strongly associated with iron deficiency [13]. Using multivariable Cox models, Jankowska et al. found that low hepcidin was independently associated with increased 3-year mortality among systolic heart failure (HF) patients [14]. These authors also found a gradual reduction in serum hepcidin levels during the natural course of HF, in accompaniment with depleted iron stores (low serum ferritin) and iron-restricted erythropoiesis (reduced hemoglobin, high red blood cell distribution width [RDW]).

Six GATA transcription factors have been identified in vertebrates and are divided into two subclasses based on their expression patterns. GATA-1, -2, and -3 are prominently expressed in hematopoietic cell lineages, whereas GATA-4, -5, and -6 are expressed in various mesoderm- and endoderm-derived tissues such as the heart, liver, lung, gonad, and gut [15,16]. Evidence for the role of GATA factors in the regulation of apoptosis was initially generated in studies of GATA-1-deficient precursor cells that were found to undergo apoptosis [17]. GATA-4 is a survival factor in terminally differentiated cardiomyocytes [18,19] and may be an essential component of the adaptive response of the adult heart [19]. In our previous study, we found that activation of ERK can protect against pro-apoptotic stimulants [20]. Notably, GATA-4 contains putative ERK phosphorylation sites, and suppression of the ERK/GATA-4 pathway induces cardiomyocyte apoptosis [20]. Notably, GATA-4 is enriched in cardiac tissue where it is essential for various cardiomyocyte physiological and adaptive responses. Moreover, an early event in doxorubicin (an antitumor drug)-induced cardiotoxicity is GATA-4 depletion, which in turn results in cardiomyocyte apoptosis [18,19]. GATA-4 has also been shown to upregulate transcription of the anti-apoptosis genes Bcl-2 and Bcl-X_L_ in cardiomyocytes [20,21]. Thus these studies support that GATA-4 plays a central role in regulating the survival of cardiomyocytes through regulation of apoptosis.

Bcl-2 protein family members are best characterized proteins for their direct involvement in the regulation of apoptosis [22]. Bcl-2 and its closest homologues, Bcl-xL and Bcl-w, potently inhibit apoptosis in response to many cytotoxic insults. Bax and Bak, on the other hand, are well known proapoptotic members of the Bcl-2 protein family. Conditions that induce myocardial stress cause complex alterations in levels of Bcl-2 family proteins [23]. Cardiac Bcl-2 gene expression has been shown to be regulated by GATA-4 both *in vitro* and *in vivo*[21]. During apoptosis, the 14 members of the caspase family in mammals are produced in cells as catalytically inactive zymogens and must undergo proteolytic activation. Caspases involved in apoptosis are generally divided into two categories: the initiator caspases, which include caspase-2, -8, -9, and -10, and the effector caspases, which include caspase-3, -6, and -7 [24].

Cardiomyocyte apoptosis can result in a loss of contractile tissue, compensatory hypertrophy of myocardial cells, reparative fibrosis, and heart failure. Bulvik et al., showed that myocardial protection by ischemic preconditioning (IPC) is mediated by a transient 'iron-signal' in concert with ferritin accumulation [25]. Our recent cross-sectional observational study in chronic kidney disease (CKD) patients [26] demonstrated that after adjustment for BMI, age, anemia, lipid profiles and blood pressure, there was a negative correlation between serum hepcidin and left ventricular mass. However, the knowledge of the hepcidin’s impact on cardiomyocytes from previous studies is limited.

Thus, we postulate that changes to hepcidin levels may regulate the apoptotic pathway of cardiomyocytes following oxidative stress. To test this hypothesis, human cardiomyocytes were cultured and treated with ferrous iron, and the effects of siRNA-mediated knockdown of hepcidin, as well as possible underlying signaling pathways of cardiotoxicity, were examined. In our knowledge, this is the first study to transfect human cardiomyocytes with hepcidin siRNA and understand the effect this has on GATA-4.

## Methods

The study was approved by the Institutional Review Board of the Changhua Christian Hospital, Taiwan. Under the approval of the Institutional Review Board of the China Medical University Hospital, Taiwan, human cardiomyocytes were obtained from myocardial ventricular resections of patients who underwent cardiac surgery, as described previously [20]. All subjects gave written and informed consent to participate.

### Cardiomyocyte culture

Human cardiomyocytes were cultured for 8 days, during which the culture medium was replaced every third day. Endogenous CAPON protein expression in cultured cardiomyocytes was detected by immunofluorescent staining and confocal microscopy [20]. We also measured electrophysiological characteristics of cultured cardiomyocytes, including action potential duration (APD) and peak L-Type calcium current (IcaL) [27]. Both APD and peak L-Type calcium current (IcaL) were APD_10_, APD_50_, APD_75_ and APD_90_: 95.4 ± 10.6, 289.2 ± 15.6, 308.2 ± 15.4, and 318.4 ± 16.4 ms, respectively, with a peak IcaL density of -10.2 ± 0.9 pA/pE at + 10 mV (n = 6) [20]. Cultured rat cardiomyocytes incubated with 20 μM iron displayed hypertrophy, while cardiomyocyte necrosis occurred at a dose of 100 μM iron [28]. Cultured human cardiomyocytes were treated with ferrous iron (500 microg/mL, 18 h) then transfected with siRNA against hepcidin. Forty-eight hours later, cellular lysates were prepared as previously described [29], and proteins were resolved on SDS-polyacrylamide gels and transferred to Immobilon polyvinyldifluoride (PVDF) membranes. Membranes were blocked with 4% (w/v) BSA for 1 h at 22.2°C and probed with rabbit anti-human Ab against hepcidin (Abcam, Abgent, San Diego, CA), Akt (Cell Signaling Technology, Beverly, MA), Caspase 8 (Millipore Corp., Billerica, MA, USA), pro-caspase 9 (Cell Signaling Technology), Bcl-2 (Santa, Cruz, CA), Bcl-xL (Santa Cruz) and β-actin (Sigma) (1/1000) for 1 h at room temperature. Protein expression was visualized by ECL using Kodak X-OMAT LS film (Eastman Kodak, Foster City, CA, USA). Quantitative data were obtained using a computing densitometer and Image Quant software.

### Bromodeoxyuridine (BrdU) assay

Cardiomyocyte proliferation was determined using the BrdU assay. Briefly, in a 96-well microplate, 2 × 10^5^ cardiomyocytes/well were incubated in 100 μL of culture media and exposed to different concentrations of ferrous iron, or Doxorubicin (DOXO, 0.5 μM) as a positive control. Our preliminary data (not shown) for the time course experiment indicated that the incubation time for the maximal effect of ferrous iron on human cardiomyocytes was 18 h. Cells were further incubated with 10 μL of 1 mM BrdU/mL and fluorescent anti-BrdU antibody for 15 min (BrdU Flow kits, BD Pharmingen, San Jose, CA, USA), then resuspended for flow cytometry analysis.

### Apoptosis assays

#### Flow cytometry

Apoptosis was quantitatively gauged by detecting phosphatidylserine exposure on cell membrane with Annexin V staining [20]. Cells were simultaneously stained with Annexin V-FITC (25 ng/ml; green fluorescence, R&D Systems, Minneapolis, MN) and dye exclusion (propidium iodide, 20 mg/ml, red fluorescence). Data were obtained by flow cytometry analysis with FACS-SCAN (Becton-Dickinson, Heidelberg, Germany) FACS Canto in cell populations from which debris was gated out and analyzed.

#### Comet assay

The comet assay (single-cell gel electrophoresis) is an uncomplicated and sensitive method for measuring deoxyribonucleic acid (DNA) strand breaks in eukaryotic cells. Cardiomyocytes (approximately 5 × 10^3^ cells/mL) were incubated with DOXO or phosphodiesterase (PDE) during peritonitis for 24 h at 37°C, isolated, then examined for DNA damage using the comet assay as previously described [30]. Briefly, treated cells were embedded *in situ* in 1% (w/v) agarose, then placed in lysis solution for 30 min. Cell nuclei were subsequently separated by electrophoresis for 20 min at 1 V/cm, followed by staining with PI and visualization under a fluorescence microscope.

### RNA isolation and reverse transcription

RNA was extracted from cells using RNAzol B (Tel-Test, Inc., Friendswood, Tex.), and was then converted to cDNA by reverse transcription. Samples were stored at -70°C until ready for analysis by PCR.

### Real-time PCR with SYBR green

For real-time PCR, 5 μL of cDNA (1–10 ng) was mixed with SYBR green PCR core reagent or master mix reagent (Applied Biosystems, CA, USA). The thermal cycling conditions were determined according to the rules of the ‘Thermal cycling parameters for primer optimization’. Each RNA sample was also analyzed for β-actin expression, which served as an internal control for correcting relative specific gene expression levels. Primers were designed using Primer Express Primer Design software, and are as follows:

Bcl-2: sense primer: ATGTGTGTGGAGAGCGTCAA

antisense primer: ATCACCAAGTGCACCTACCC

Bcl-x_L_: sense primer: ACAGCAGCAGTTTGGATGC

antisense primer: TGGGATGTCAGGTCACTGAA

GATA-4: sense primer: AGCTCCTTCAGGCAGTGAGA

antisense primer: CTGTGCCCGTAGTGAGATGA

β-actin: sense primer: CAGGTATGCACCCAGAGTGA

antisense: GATATGGAGAAGATTTGGCA

According to the amplification plot, the cycle number over the threshold equals the Ct value. The Ct value of the non-template control was 45. The relative expression ratio among untreated RNA and different RNA samples could be thus calculated by 2-Ct.

### Western blotting

For western blotting, 10–50 μg of protein extracts were separated using 10–12% SDS-polyacrylamide gel electrophoresis, transferred to nitrocellulose (PVDF) membranes, then blocked overnight with 1 × tris buffered saline buffer containing 5% (w/v) skim milk. Membranes were next incubated with optimal concentrations of primary antibodies: anti-GATA-4 mAb (Abcam), and anti-β actin mAb (Sigma) all diluted in blocking buffer. Membranes were washed and then incubated with appropriate secondary antibodies (goat anti-mouse mAb conjugated with HRP), and visualized using the enhanced chemiluminescence detection kit (Perkin Elmer, MA, USA). The antibodies for GATA-4 (1:100) and Bcl-X_L_ (1:100) were purchased from Santa Cruz (CA, USA) while Bcl-2 (1:1000) was purchased from Cell Signaling (CA, USA).

### siRNA transfection

Two pairs of small-interfering RNAs (siRNA1 and siRNA2) were synthesized by Invitrogen Life Technology (Invitrogen). siRNA2 was employed to knock-down hepcidin levels and siRNA1 was injected s the negative control. Cultured human cardiomyocytes were transfected with each siRNA (20 μM) using Lipofectamine RNAiMAX (Invitrogen) as per the manufacturer’s instructions. After 48 h, protein was extracted for western blot analysis.

### Statistical analyses

Values are expressed as the mean ± standard deviation (SD). Parameters between groups were compared using the Jonckheere Terpstra or Kruskal-Wallis test for non-parametric continuous data, or the independent t-test for parametric data. Statistical analysis was performed via SPSS for Windows software, version 15.0. A P-value < 0.05 was considered statistically significant.

## Results

### Ferrous iron induces cell death in human cardiomyocytes

**Figure 1:**
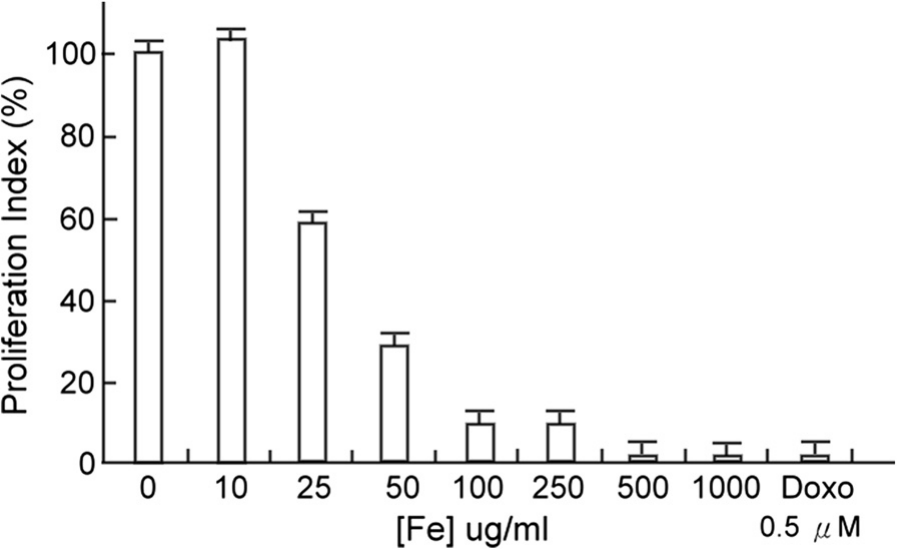
**Cell proliferation of human cardiomyocytes treated with different concentrations of ferrous iron.** Doxorubicin (DOXO, 0.5 μM) was used as a positive control. Data are expressed as the mean ± SD of six independent experiments.

Cardiac cell death is believed to be a major contributor in the development and progression of myocardial dysfunction [31]. To assess whether ferrous iron treatment induces cardiac death, cell viability of cardiomyocytes treated with ferrous iron, or DOXO as a positive control, was evaluated by BrdU flow cytometry. The BrdU assay revealed that ferrous iron induced human cardiomyocyte cell death in a dose-dependent manner (Figure 1). As expected, DOXO also induced cardiotoxicity.

### Ferrous iron induces apoptosis in human cardiomyocytes

**Figure 2:**
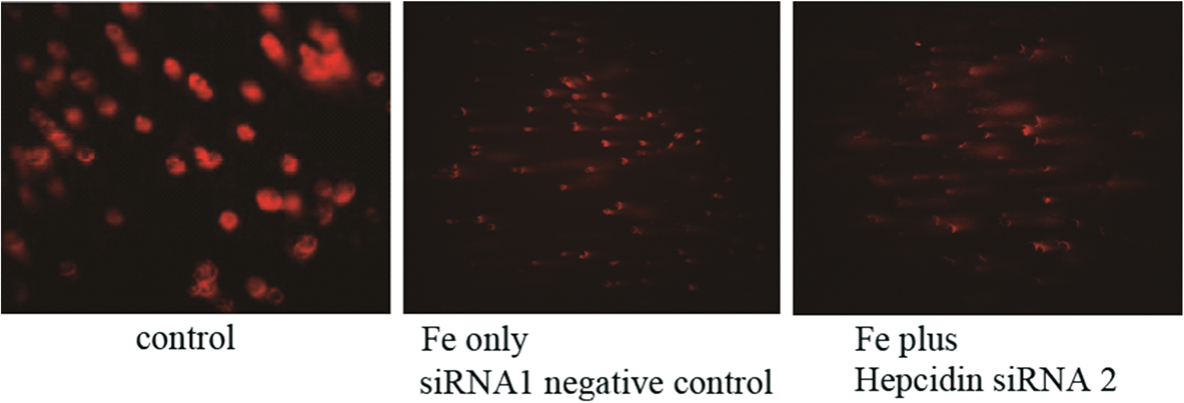
**Comet assay: effect of silencing hepcidin on ferrous iron-induced cardiomyocyte apoptosis.** siRNA2 was employed to knock-down hepcidin levels and siRNA1 was injected as a negative control. Cultured human cardiomyocytes, pretreated with ferrous iron (500 microg/mL, 18 h), were transfected with each siRNAs (20 μM, 48 h). DNA damage was then determined by the comet assay. Ferrous iron-free treated cells were used as the control.

To further explore whether ferrous iron accumulation induces human cardiomyocyte apoptosis, we assessed apoptotic cell death using Annexin V-FITC and flow cytometry. As above, DOXO (0.5 μM) was used as a positive control. After cell incubation with ferrous iron for 18 h, apoptosis was observed. Higher concentrations of ferrous iron resulted in greater numbers of damaged cells (Table 1). Using the comet assay (Figure 2), we also monitored the extent of cardiomyocyte apoptosis following pretreatment with ferrous iron and more severe cardiomyocyte apoptosis damage following transfection with siRNA2 to knockdown hepcidin expression.

**Table 1:** Effect of different doses of ferrous iron on apoptosis in cultured human cardiomyocytes

**Ferrous Iron (μg/ml)**	**% apoptosis (mean ± SD)**
0	1.0 ± 2.2
10	1.1 ± 2.3
50	4.2 ± 2.1
100	7.6 ± 2.5
250	8.4 ± 2.3
500	11.2 ± 2.1
Positive control: DOXO (0.5 μM)	10.1 ± 2.2

Positive control: medium + DOXO (0.5 μM): 10.1 ± 2.2%.

Cultured human cardiomyocytes were incubated with medium alone or in combination with ferrous iron at the indicated concentrations for 18 h to reach the maximal effect of ferrous iron. Medium plus DOXO (0.5 μM) was used as a positive control. Following the maximal effect of ferrous iron, cells were stained with annexin V-FITC and apoptosis analysis was determined by FACS. Each value represents the mean ± SD from six independent experiments.

### Ferrous iron suppresses GATA-4 expression in human cardiomyocytes

**Figure 3:**
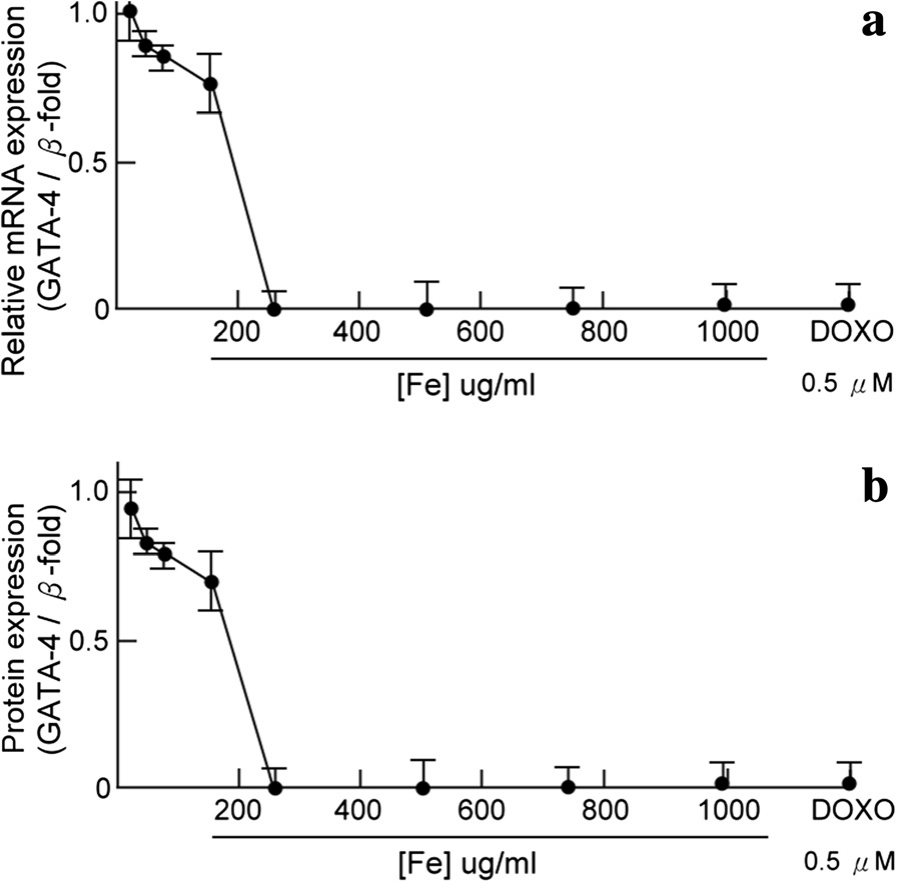
**Effects of ferrous iron on GATA-4 m-RNA and protein levels in cardiomyocytes.** GATA-4 mRNA expression was decreased in a dose-dependent manner after incubation with ferrous iron (Figure 3**a**). Western blots of nuclear GATA-4 protein expression also showed a dose-dependent decrease in response to ferrous iron in cardiomyocytes (Figure 3**b**). Cultured human cardiomyocytes were treated with different concentrations of ferrous iron. Doxorubicin (DOXO, 0.5 μM) was used as a positive control. Data are normalized to β-actin, and are expressed as the mean ± SD of six independent experiments.

The transcription factor GATA-4 is a well-known specific myocardial survival factor [32]. To characterize the mechanisms underlying ferrous iron-induced apoptosis in human cardiomyocytes, mRNA expression and protein levels of GATA-4 were measured. GATA-4 mRNA expression was decreased in a dose-dependent manner after incubation with ferrous iron (Figure 3a). Western blots of nuclear GATA-4 protein expression also showed a dose-dependent decrease in response to ferrous iron in cardiomyocytes (Figure 3b).

### Silencing of hepcidin induced apoptosis in iron-induced human cardiomyocytes

**Figure 4:**
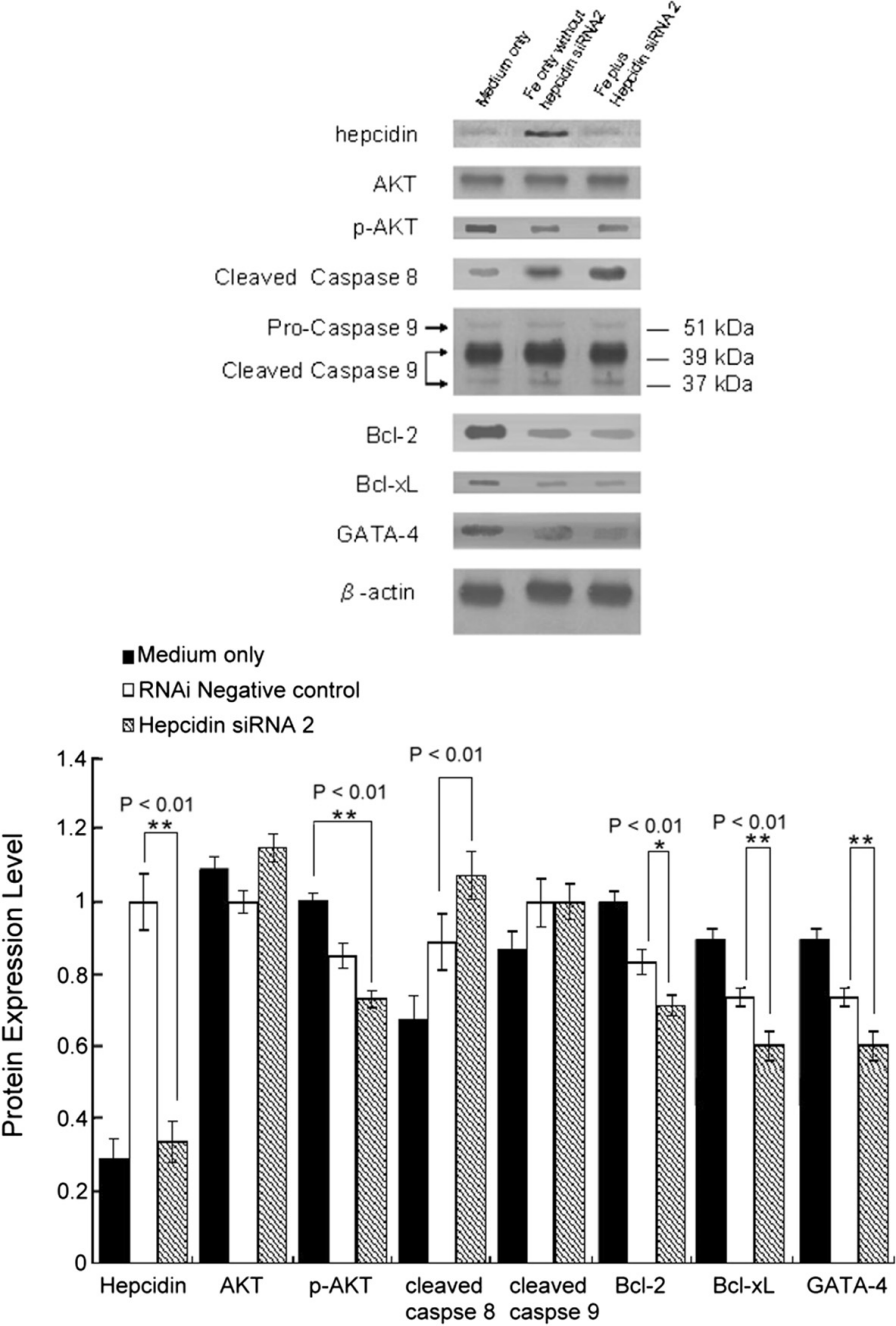
**Hepcidin knockdown by siRNAs alters the apoptotic protein expression profile.** siRNA2 was employed to knock-down hepcidin levels. Cultured human cardiomyocytes were transfected with each siRNAs (20 μM). After 48 h, proteins were extracted for western blot analysis. The protein expression of caspase-8, Bcl-2, Bcl-xL and phosphorylated Akt, were measured by western blotting. Cultured human cardiomyocytes were pretreated with ferrous iron (500 mg/mL) only (lane 2) and in combination with siRNA2 for silencing hepcidin (lane 3). Cells cultured in ferrous iron-free medium were also analyzed (lane 1). Quantitative densitometry was performed and a histogram was developed showing protein expression relative to total protein. Data are expressed as the mean ± SD of six independent experiments. P-values are indicated.

To further explore the mechanisms of ferrous iron-induced apoptosis, other well-known apoptotic markers were examined by western blotting. After cardiomyocyte treatment with ferrous iron (500 mg/mL, 18 h; middle column of Figure 4), in comparison to non-iron medium, there was increased expression of both hepcidin and cleaved caspase 8, and a significant decrease in Bcl-2, Bcl-xL, phosphorylated Akt and GATA-4 expression levels. Following hepcidin siRNA2 transfection and treatment with ferrous iron (right column of Figure 4), hepcidin protein returned to non-iron treated levels. Meanwhile, the synthesis of cleaved caspase 8 also significantly increased and protein expression levels of Bcl-2, Bcl-xL, phosphorylated Akt and GATA-4 were significantly suppressed when compared with the non-iron medium group.

Following treatment with ferrous iron and siRNA2 (right column of Figure 4), cleaved caspase 8 levels were higher and protein levels of Bcl-2, Bcl-xL, phosphorylated Akt and GATA-4 were significantly lower than treatment with ferrous iron only (middle column of Figure 4). There was no significant difference among the conditions with respect to caspase 9 protein levels.

## Discussion

In the present study, we have demonstrated for the first time that transfection of human cardiomyocytes with hepcidin siRNA can downregulate anti-apoptotic GATA-4/Bcl-2. By contrast, hepcidin siRNA augmented protein expression of pro-apoptotic caspase 8. This finding was consistent with our earlier published clinical study which found that there was a significant negative correlation between serum hepcidin levels with either left ventricular mass (LVM) or the left ventricular mass index (LVMI) in CKD patients [26]. We also found that ferrous iron-induced human cardiomyocyte apoptosis was associated with decreased protein expression of GATA-4, Bcl-2, and Bcl-xL, and phosphorylated Akt. In addition, ferrous iron increased the expression of hepcidin and caspase 8 in human cardiomyocytes.

Iron ions stored within the ferritin molecule are redox-inactive and cannot catalyze the generation of free radicals [33]. By contrast, excessive extracellular iron in heart muscle is thought to induce the formation of ROS. Therefore, ferritin is assumed to play an important cytoprotective role against free radical formation by controlling free cytosolic iron content [34]. Moreover, it has been shown that ROS formation, amplified by labile and redox-active iron ions, increases the susceptibility to cardiac injury and cell death [35]. Indeed, in the BrdU cell viability study, we showed that ferrous iron-induced human cardiomyocyte cell death was dose-related. Consistent with our finding, Munoz et al. showed that different iron concentrations exhibit diverse effects on cultured cardiomyocytes, whereby either its excess or deficiency inflicts myocardial damage [28].

There is convincing evidence that apoptosis contributes to the progression of heart failure [36]. Inflammatory mediators and cytokines have been cited as playing a role in cardiomyocyte apoptosis and clinical cardiac dysfunction [37]. More recently, experiments demonstrated that anthracycline cancer chemotherapeutic agents, such as daunorubicin and doxorubicin, cause cardiomyopathy and also induce cardiac myocyte apoptosis [38]. Using doxorubicin as a positive control, we demonstrated that greater ferrous iron levels resulted in more apoptosis in human cardiomyocytes. Cardiogenesis is known to be enhanced in embryonic stem cells overexpressing GATA-4, while its depletion results in apoptosis [39]. Survival factors of cardiomyocytes, such as hepatocyte growth factor and endothelin-1, activate GATA-4 to protect the heart against oxidative stress [19]. Our results also show that exogenous iron can downregulate the expression of GATA-4 mRNA and protein levels in a dose-dependent manner.

Importantly, following human cardiomyocyte pretreatment with ferrous iron, we noted that the expression levels of hepcidin and caspase 8 increased, while those of Bcl-2, Bcl-xL, and phosphorylated Akt significantly decreased. From the study, we suggest that the pro-apoptotic pathway contributes to the iron-associated cardiomyopathy. In the heart, hepcidin is an intrinsic cardiac hormone which has a predominantly local effect [40]. Moreover, studies in the ischemic and non-ischemic myocardium after myocardial infarction showed particular upregulation of hepcidin [41]. The authors speculated that upregulation of hepcidin may reduce iron toxicity and thus infarct size expansion in an infarcted heart [41]. Our present findings seem to be consistent with these assumptions that beyond iron regulation, hepcidin expression in the heart could impact on heart function. In the present study, however, we did not link Akt activation and the GATA-4-Bcl-2/Bcl-xL pathway.

Island et al. reported that GATA-4 may participate in the control of hepcidin expression and that the alteration of its expression could contribute to the development of iron-related disorders [42]. Previous research [43] has shown that overexpression of Bcl-2 attenuates myocardial apoptosis. Similarly, in an *in vivo* study we found that hepcidin may be involved in cardioprotection [26]. In the present study, we found that the combined treatment of human cardiomyocytes with hepcidin siRNA and ferrous iron led to the increased synthesis of cleaved caspase 8, while that of hepcidin, as well as other anti-apoptotic proteins, were significantly suppressed. These findings indicate that silencing hepcidin could downregulate anti-apoptotic protein synthesis and provoke cardiomyocyte death. Indeed, evaluation of DNA damage by Comet assay confirmed the negative impact of hepcidin siRNA on cardiomyocytes.

In addition, following silencing of hepcidin mRNA expression, the synthesis of caspase 8 was enhanced. By contrast, caspase 9 levels did not significantly change. As known, the extrinsic apoptotic pathway is mediated by caspase 8, which is first activated by the death receptors and, in turn, activates caspase 3. In the intrinsic pathway, caspase 9 is first activated by mitochondria-released cytochrome C [44]. Based on these observations, we propose the extrinsic apoptotic pathway could be the target for cardioprotection by hepcidin.

There were several limitations in our studies: (1) Given that only caspase 9 was monitored, it is difficult to attribute the observed apoptosis to the extrinsic pathway alone. To rule out any involvement of the intrinsic pathway, we need to monitor at least cytochrome c release or inhibit caspase 8 activation to determine if cell death could be completely inhibited. (2) The knockdown of hepcidin resulted in an increase in apoptosis, but suggested that hepcidin played a protective role. If we could show that over-expression of hepcidin resulted in an increase in Bcl-2, GATA-4, Bcl-xl etc. and that would greatly enhance our work.

In conclusion, this study demonstrates that hepcidin acts as a novel survival factor for human cardiomyocytes through the regulation of GATA-4/Bcl-2 and the extrinsic apoptotic pathway. These results complement our earlier clinical findings that serum hepcidin is significantly negatively correlated with LVM and LVMI [26].

## Competing interests

The authors declare that they have no competing interests.

## Authors’ contributions

CY Lin and CC Chang conceived of the study; YP Hsieh, CH Huang and CC Chang participated in the design and coordination of the study; YP Hsieh and CY Lee carried out the experiments, analyzed the data; YP Hsieh and CC Chang wrote the manuscript; CY Lin contributed to the guidance of experiments; and CC Chang read the manuscript and revised it for important intellectual content. All authors have read and approved the final manuscript.
